# Post‐operative pain behaviour associated with surgical castration in donkeys (*Equus asinus*)

**DOI:** 10.1111/evj.13306

**Published:** 2020-07-08

**Authors:** Maria Gláucia Carlos de Oliveira, Stelio P. L. Luna, Talyta Lins Nunes, Paulo R. Firmino, Amara Gyane A. de Lima, Josiel Ferreira, Pedro H. E. Trindade, Raimundo A. B. Júnior, Valéria Veras de Paula

**Affiliations:** ^1^ Universidade Federal Rural do Semi‐Árido (UFERSA) Mossoró, Rio Grande do Norte Brazil; ^2^ São Paulo State University (Unesp) Botucatu Brazil; ^3^ Bahia Federal University (UFBA) Salvador Brazil

**Keywords:** horse, well‐being, donkeys, analgesia, castration

## Abstract

**Background:**

Recognising pain in donkeys is challenging because they are stoic.

**Objectives:**

To identify the responses of donkeys before and after surgical pain.

**Study design:**

Prospective, short‐term longitudinal pre‐ and post‐intervention observations.

**Methods:**

Forty adult donkeys underwent surgical castration after sedation with intravenous (IV) xylazine, induction with guaiphenesin/thiopental IV and maintenance of anaesthesia with isoflurane and local anaesthetic blockade. Four hours after recovery from anaesthesia, flunixin meglumine 1.1 mg/kg, dipyrone 10 mg/kg and morphine 0.2 mg/kg IV were administered. Behavioural responses exhibited by the animals housed in individual stalls were recorded in four 30‐min videos: before castration (M0), and 3.5‐4.0 hours (M1), 5.5‐6.0 hours (M2) and 23.5‐24.0 hours after recovery from anaesthesia (M3). To exclude the influence of insects, the behaviour of six apparently pain‐free donkeys was compared with and without the presence of faeces and urine in the stall.

**Results:**

When compared with presurgical baseline behaviours (M0), after surgery (M1) donkeys raised their pelvic limbs more (*P* = .003). When compared with M1, after analgesia (M2), the median frequencies of ear movements (44 vs 16; *P* < .001), head shaking (7 vs 1; *P* < .001), head turning (5 vs 0; *P* < .001) and lifting of the both limbs (7 vs 0; *P* = .008) decreased; feeding (0 vs 29; *P* < .001) and water intake (0 vs 0, range 0‐1 vs 0‐7; *P* = .05) increased. The dirty stall increased tail (53 vs 80; *P* = .03), head (16 vs 30; *P* = .03) and ear movements (50 vs 78; *P* = .04).

**Main limitations:**

The dirty stall and presence of insects possibly contributed to the expression of behaviours unrelated to pain.

**Conclusion:**

Lifting the pelvic limbs was the only specific pain behaviour after castration in donkeys. Analgesia restored appetite and water intake and reduced the frequency of head shaking and turning, ear movement and lifting the limbs. Tail, head and ear movements are unspecific responses related both to pain and a dirty stall, and are confounding factors when pain is assessed in donkeys in the presence of insects.

## 1 INTRODUCTION

Donkeys are used as a labour force in agriculture and transport by the poorest populations in developing countries.^1^, ^2^, ^3^ Identifying behavioural changes in this species can be challenging.^4^ Their stoic behaviour, characterised by a lack of clear expressions of pain, coupled with poor knowledge of their normal habits, make it difficult to understand their pain conditions and hamper the establishment of adequate analgesic treatments.^1^ When evaluating pain behaviour in donkeys, it has been observed that donkeys do not express pain in an obvious way, possibly due to greater tolerance of adverse conditions or the inability of the observer to interpret the signs.^1^

To date, there are no studies that use a standardised pain model to evaluate pain‐related behaviour and develop a validated instrument to assess pain in donkeys. There is an ethogram for donkeys used for transportation of loads^5^; however, these animals were affected by several painful clinical conditions, without standardisation of the painful stimulus. The same group reported that analgesia relieved the expression of pain‐indicating behaviours.^6^

In horses, castration produces pain that can persist for several days and, therefore, requires adequate analgesics.^7^ Based on the hypothesis of this study that donkeys exhibit particular behavioural expressions of pain, our objective was to identify and quantify the responses shown by donkeys subjected to pain induced by surgical castration. In addition, the effect of a dirty stall and, therefore, the presence of insects, was evaluated on the normal behaviour of the donkeys.

## 2 MATERIALS AND METHODS

Forty adult intact male donkeys weighing 120 ± 13 (87–133) kg and approximate age 6.4 ± 3.1 (2–14) years from an Animal Protection Association were used. For study inclusion, the donkeys were required to allow human approach and halter placement and be apparently healthy based on physical and laboratory examinations. Physical examination included cardiorespiratory and abdominal auscultation, capillary refill time, mucous membrane colour, faecal characteristics and evaluation of lameness. Laboratory examinations included haemogram, urea, creatinine, total protein and aspartate and alanine aminotransferase plasma concentrations.

The animals were dewormed with 0.2 mg/kg bodyweight (bwt) of ivermectin intramuscular (IM) (Ivomec^®^) and vaccinated against rabies (Rai‐Vet Líquida^®^) 4 weeks before the start of the study. They were housed in groups of 5 in a 17 × 13 m outdoor area with shade. Donkeys were fed with 7.5 kg/100 kg bwt of Napier grass (*Pennisetum purpureum)* and 1.1 kg/100 kg bwt of concentrate (ground corn, soybean, wheat bran, common salt and calcitic limestone) twice daily and had *ad libitum* access to water. The period of acclimatisation to the new environment and human interaction was 4 weeks.

One day before the surgical procedure, the animals were transferred to an individual stall (3 × 7 m) for acclimation. Feed and water were withheld for 12 and 6 hours respectively. After sedation with 0.5 mg/kg bwt of xylazine (Equisedan^®^) IV, anaesthesia was induced with 100 mg/kg bwt of guaiacol glyceryl ether (GGE PPU^®^) and 5 mg/kg bwt of thiopental (Thiopentax^®^) IV. Anaesthesia was maintained with isoflurane (Isoforine^®^) in 10 mL/kg/min of O_2_. Local anaesthetic (15 mL/testicle of 2% lidocaine, Lidovet^®^) was infiltrated into each spermatic funiculus and the proposed incision line. Surgical castration was performed by the same surgeon using an open technique.^8^ At the end of surgery, the donkeys received tetanus antitoxin (Lyophilized Anti‐tetanus Serum^®^) and 30,000 IU/kg bwt of sodium penicillin (Pentabiotic^®^) IM. The donkeys were returned to the same stall as prior to the surgical procedure. Grass and water were offered 2 hours after recovery from general anaesthesia. Recovery time was recorded from the end of isoflurane administration until the donkey stood.

To observe the effect of intervention analgesia on pain‐related behaviours, the donkeys received analgesia only 4 hours after recovery from anaesthesia. Analgesia was composed of 1.1 mg/kg bwt of flunixin meglumine IV (Flunixin UCB^®^), 10 mg/kg bwt of dipyrone IV (Febrax^®^) and 0.2 mg/kg bwt of morphine IV (Dimorf^®^), and was repeated every 24 hours for 3 days. The surgical wound was treated twice a day for 3 days with silver sulfadiazine (MaxPrata^®^). Videos of the donkeys’ behaviour, lasting 30 minutes, were recorded in the individual stall during the following periods: before the surgical castration in the morning (M0 – baseline; before stall cleaning), between 3.5 and 4.0 hours after recovery from anaesthesia (M1 – pain; after stall cleaning), between 5.5 and 6.0 hours after recovery from anaesthesia (M2 – 1.5 and 2.0 hours after the administration of analgesics) and between 23.5 and 24.0 hours after recovery from anaesthesia on the following morning (M3 – 24h; before stall cleaning).

Four donkeys were castrated in a pilot study to provide a list of behaviours to be combined with the list of behaviours previously reported for donkeys suffering pain^1^ (Table S1). The same person assessed all videos from the pilot and the main study to record and quantify the responses exhibited by the donkeys before and after surgery. The time donkeys remained standing, moving or eating was recorded. A greater number of insects were observed in the mornings before stall cleaning (M0, baseline and M3, 24 hours) compared with after stall cleaning (M1, pain and M2, after analgesia). This possible confounding factor was detected after the main study had been completed. To investigate this possible limitation, considering that the greater presence of insects at M0 (baseline) and M3 (24 hours) might change the behavioural responses before and after surgery, a parallel study was performed to evaluate the influence of cleaning the stall on responses exhibited. Six donkeys were observed in two situations: (a) stall with (before morning cleaning) and (b) without faeces and urine (after morning cleaning), factors that influence the higher or lower number of insects respectively. The animals were videoed in the morning for 30 minutes immediately before and after stall cleaning.

Statistical analyses were performed using R software in the integrated development environment RStudio (Version 1.0.143 – ^©^ 2009‐2016), considering an α of 5%. The normality of each variable at each time period was evaluated by box graphs and histograms. According to that, data were considered non‐normal. To evaluate increases or decreases in responses over time, a Friedman test was performed with *P*‐values corrected using the Bonferroni procedure. To verify the influence of a dirty stall on the behaviour of donkeys, the responses exhibited in the presence of faeces and urine were compared with responses in the clean stall, applying the paired and two‐tailed Wilcoxon test.

## 3 RESULTS

The duration of the surgery was 34 ± 6 (28–40) minutes and the donkeys were standing 45 ± 7 (38–52) minutes after the end of anaesthesia. Twenty‐five behaviours were observed during the 80 hours of video analysis of the 40 donkeys. Two animals had some bleeding from scrotal wounds after castration, which stopped 10 minutes after surgery. Nine animals had preputial and scrotal oedema 2‐3 days after surgery, subsiding in 4 days. There were no other post‐operative complications during the period the donkeys were videoed.

The median and range of the frequency of each behaviour occurred in the four time periods are presented in Table 1. During the observation period the animals were either standing in different places in the stall, moving about the stall or eating (Table 1). Eating behaviour and water intake increased after analgesic treatment (M2) and the time spent in locomotion did not change during the 4 time periods.

**Table 1:** Median and range (minimum – maximum) of the frequency or duration of time (minutes) for each behaviour of donkeys observed on the video recording, before and after surgical castration

Behaviours	M0^§^		M1^#^		M2^#^		M3^§^	
	Med	Range	Med	Range	Med	Range	Med	Range
Ear movement	55^a^	0‐110	43.5^b^	6‐154	16^c^	1‐43	52.5^a^	18‐114
Eating^‡^	0^bc^	0‐2.1	0^c^	0‐2	28.7^a^	0‐30	0^b^	0‐14.3
Flexes thoracic limbs	4^ab^	0‐46	4^a^	0‐50	0^b^	0‐14	3^a^	0‐39
Head shaking	11.5^a^	1‐225	6.5^b^	0‐89	1^c^	0‐12	9^b^	0‐150
Head turning	3^ab^	0‐37	4.5^a^	0‐37	0^b^	0‐41	4.5^a^	0‐77
Lifts pelvic limb	2^b^	0‐55	7^a^	0‐100	0^b^	0‐28	2^b^	0‐19
Scratching†	3^a^	0‐12	1.5^ab^	0‐20	1^b^	0‐9	2^ab^	0 – 16
Standing at the back of the stall^‡^	1.7^a^	0‐30	1.66^a^	0‐30	0^b^	0‐26.7	0.3^a^	0‐30
Standing at the front of the stall^‡^	9^a^	0‐29	4.75^a^	0‐30	0^b^	0‐30	11.3^a^	0‐30
Standing at the stall door^‡^	1.8^ab^	0‐20	0^bc^	0‐9	0^c^	0‐23	2^a^	0‐30
Tail swishing	76.5^a^	17‐615	43^c^	1‐210	29.5^bc^	0‐394	54.5^ab^	2‐300
Water drinking	0^b^	0‐4	0^b^	0‐1	0^a^	0‐7	0^ab^	0‐4
Yawn/Flehmen response	0^a^	0‐42	0^ab^	0‐4	0^b^	0‐3	0^ab^	0‐5

Note

Each period of time corresponded to 30‐minute video clips obtained from 40 donkeys before surgical castration (M0), between 3.5 and 4 h after recovery from anaesthesia (M1), between 5.5 and 6 h after recovery from anaesthesia and from 1.5 to 2 h after analgesic rescue (M2), and between 23.3 and 24 h after recovery from anaesthesia (M3).

^a,b,c^ different letters indicate a statistical difference between time periods, where a > b > c; Med = Median.

^†^ includes scratching the thoracic limb with the head, scratching the head on the water trough, scratching the head against the wall, scratching the head with a pelvic limb or scratching the flank with the head.

^‡^ time in minutes of the activities.

^§^ dirty stall.

^#^ clean stall. Comparisons were performed with the Friedman test (*P* < .05). Data from the behaviours braying, defecation, exposes penis, extends pelvic limbs, flank watching or staring, interacts with animal in another stall, investigates the stall door, kicking, movement around the stall, pawing at the ground, standing in the middle of the stall and urination were not included because there were no significant changes and median values were zero or close to zero (data are shown as support material).

Lifting pelvic limbs occurred more in M1 (pain) than the other time periods (*P* ≤ .003). Flexing the thoracic limbs and turning the head occurred significantly fewer times during M2 (after analgesia) compared with M1 (pain) (*P* = .008 and *P* < .001 respectively).

Shaking the head or tail and ear movements were observed more frequently before the surgical castration (M0) compared with M1 (pain) and M2 (after analgesia) (*P* ≤ .01 in all cases). There was a trend for these behaviours to decrease after surgery and increase 24 hours after recovery from anaesthesia (M3). However, a dirty stall influenced the expression of these behaviours (Table 2).

**Table 2:** Median and range (minimum – maximum) of the behaviours observed in six donkeys in the stall before the morning cleaning (dirty stall) or after the morning cleaning (clean stall)

Behaviours	Dirty Stall		Clean Stall	
	Med	Range	Med	Range
Defecation	0	0‐1	0	0‐0
Ear movement	78^a^	56‐199	49.5^b^	33‐121
Expose penis	0	0‐1	0	0‐1
Flexes thoracic limbs	6.5	0‐11	5	1‐10
Head shaking	29.5^a^	17‐66	15.5^b^	10‐26
Head turning	17.5	10‐26	16.5	7‐22
Investigates door of stall	0	0‐0	0	0‐1
Look for food	0	0‐2	1	0‐15
Scratches flank with head	2	0‐6	0	0‐2
Scratches head on wall	0	0‐4	0	0‐4
Scratches head with pelvic limb	0	0‐1	0	0‐0
Scratches thoracic limb with head	3	1‐6	1	0‐7
Tail swishing	79.5^a^	39‐151	53^b^	14‐98
Urination	0	0‐1	0	0‐1
Water drinking	0	0‐1	0	0‐1
Yawn/Flehmen response	0	0‐4	0	0‐1

^a,b,c^Different letters indicate statistical difference between dirty and clean stall, where a > b; Wilcoxon, α of 5%.

Sample size was not calculated before the beginning of the study, as no other data were available from previous studies; however, the power is likely to be sufficient to detect reasonable effects.

## 4 DISCUSSION

This is the first study to describe the behaviours presented by donkeys with acute pain after a standard nociceptive stimulus such as surgical castration. We identified behaviours indicative of acute pain for 24 hours after surgical castration in the donkeys. These behaviours are not easily identifiable in this species, which confirms our hypothesis that donkeys have a particular pain behaviour. Surgical castration was used as a nociceptive model since it is a standardised surgical stimulus and the most common in the species, as well as for which there is a validated scale available with the same procedure in horses^9^ as a comparison model.

Studies aiming to define post‐operative pain recognition in animals require an experimental design that guarantees the expression of identifiable pain behaviours. These behaviours should be unrelated and not treated by the anaesthetic and analgesics used in the surgical protocol. The drugs used in this study with analgesic properties were xylazine and lidocaine. The antinociceptive effect of xylazine lasts 60 minutes^10^ and lidocaine 30‐90 minutes in horses.^11^ Therefore, the reason rescue analgesia was administered 4 hours after recovery from anaesthesia was to guarantee that the effects of these drugs had abated and pain expression would be observed. A similar approach was adopted in studies performed in other species,^9^, ^12^ which showed that the animals displayed the most intense post‐operative pain during this period.

The analgesic protocol used in this study was based on the same multimodal analgesic protocol used in horses undergoing surgical castration^9^ and with the same recommended doses reported in horses.^13^ Combining analgesic agents with different mechanisms of action can be of benefit to guarantee the effectiveness of analgesia.^14^, ^15^

Lifting the pelvic limbs was the main behaviour observed in the donkeys with pain. This behaviour has also been observed in horses after surgical castration with a high specificity and sensitivity for pain.^9^ Analgesia restored food and water intake and reduced the frequency of other behaviours observed before analgesia, such as head shaking and turning, ear movement and lifting the limbs.

The limited behavioural manifestation of pain in donkeys is probably due to the need for resistance to the adverse conditions routinely experienced by this species, making them stoic. The pain behaviours observed in this study were similar to those reported in donkeys during work,^4^ before and after administration of meloxicam during the transport of loads^6^ and with various clinical diseases.^5^ However, after surgery and before rescue analgesia, the behaviours commonly expressed in horses with abdominal pain and after surgical castration such as looking at the flank,^9^, ^16^ kicking the abdomen, moving the head^9^ and pawing at the ground^17^ were not observed. The lesser expression of these behaviours corroborates the greater subtlety of donkeys in expressing pain.^2^

Experimental studies confirm the differences in clinical pain expression between donkeys and horses, since the thermal nociceptive threshold, evaluated in a temperate region, characterised by the flexion reflex of the limb or attention to the affected area, under temperatures of 51°C, did not occur in donkeys, which led the authors not to recommend this test for the species.^18^ On the other hand, the thermal threshold of 51.4°C triggers a reaction to stimulus aversion in horses, even in a tropical climate.^19^ In this way, both clinical and experimental results demonstrate that donkeys are apparently more resistant or less expressive of discomfort in relation to horses.

From a physiological stand point, the lower expression of pain is apparently not related to a lower cortical response of pain perception between donkeys and horses. When evaluating the sensorial processing of donkeys and horses with the same nociceptive (castration) stimulus, donkeys produced similar or greater baseline responses on the electroencephalogram.^20^ An interesting outcome from these results is that there was a significant increase in the spectral edge frequency during surgical incision and emasculation, which led to a greater change in the total power of the donkey electroencephalogram, in relation to both basal values and to ponies, which suggests that donkeys "mask" the expression of pain.^20^

The reduction in head, ear and limb movements and the increase in eating and drinking behaviours after analgesia are associated with the well‐being promoted by analgesic administration. Restless behaviour has been described as a nonspecific indicator of pain in horses.^1^ In a study with donkeys,^6^ it was observed that after one dose of meloxicam the animals were more alert and investigative and lay down, dozed, closed their eyes, changed the distribution of bodyweight less frequently and maintained their head higher.

A possible limitation of this study was that it is not possible to guarantee that the donkeys were completely free of pain. Although they had a thorough pre‐operative assessments, including gait analysis, the donkeys were rescued from a highway and, therefore, it was not possible to eliminate a subclinical musculoskeletal disease because diagnostic imaging was not performed. Another limitation was that the pre‐operative behaviour assessment was performed early in the morning before the stall was cleaned and, therefore, the stall was dirty causing insects to be present. As this situation could interfere with the frequency of some responses indicative of pain, an additional study was performed to evaluate the behaviour of the animals before and after cleaning the stall and detect any unrelated pain responses. Shaking the tail and head, scratching and moving the ears were the most obvious behaviours associated with insects that might increase because of the dirty stall and promote a false‐positive result suggesting that the animals were in pain before surgery. As these behaviours are cited in the literature as indicative of pain in horses^9^, ^21^, ^22^ and donkeys,^1^ six animals were monitored by video at the same time of day that baseline measurement was performed in the main study. Evaluations were performed at baseline (dirty stall) and again after cleaning the stall.

Shaking the tail and head and the ear movements were reduced after cleaning the stall. Responses before cleaning the stall were similar to M1 (post‐castration before analgesia); therefore, were not related to post‐castration pain only. In a previous study, a dirty stall, possibly with a great number of insects, also appeared to elicit shaking the head and tail and excessive limb raising.^4^ Defensive behaviours against insects are characteristic to reduce annoyance.^23^ In horses bothered by insects, rapid rotation of one or both ears, the tail swinging from top to bottom and from one side to the other, biting, licking or rubbing any part of the body and rapid rotation of the head and neck are also observed.^24^ In this way, the act of shaking the tail in isolation should not be considered as characteristic of pain, since this behaviour may be related to restlessness.^2^ A possible effect of time of the day on these behaviours was not investigated. A control group without surgical stimulus could clarify these points.

The literature is contradictory as to whether pain alters the frequency and type of ears movements in donkeys. While some authors report that donkeys in pain move their ears more,^1^, ^6^ others report that this species moves their ears less and responds less to noises or other stimuli when they are in pain.^2^ In the present study, ear movements decreased during the period of greatest pain (M1) compared with the basal value and reduced even further after the analgesic rescue (M2). As in the clean stall, ear movement was less than in the dirty stall, apparently the reduction in this movement is more related to cleaning at M1 and to the possible effect of morphine at M2.

Both appetite and water intake were established after analgesia, which suggests that pain produces anorexia in donkeys. It was not possible to assess food and water intake before the surgery as the animals were fasting for both water and solids. Apparently, inappetence together with a lack of interest in drinking water are the first signs observed in donkeys in pain.^2^ Although some animals approach the food trough and appear to eat, they do not ingest the food; this requires observer's attention to identify anorexia. In horses with orthopaedic pain, the presence or absence of appetite, together with other variables, may also help in identifying and differentiating pain levels.^21^

Regarding the duration of the behaviours, the donkeys did not move around the stall much during any period, which indicates that this species tends to spend more time stationary and that this situation does not change with pain as previously reported.^25^ Although it has been cited that donkeys in pain tend to remain stationary with their head lowered^25^ in the current study, the animals did not exhibit head lowering at any time. Standing still is considered a sign of stress in donkeys, while horses tend to be restless. It is also unusual to observe donkeys lying down or rolling like horses with pain.^26^

It was concluded that lifting the pelvic limbs was the only specific behavioural indicator of pain after castration in donkeys. Analgesia restored appetite and water intake and reduced the frequency of head shaking and turning, ear movement and lifting the limbs. Tail, head and ear movements are nonspecific responses related both to pain and probably the presence of insects in a dirty stall.

## Conflict of interests

No competing interests have been declared.

## Ethical animal research

The study was approved by the Ethics Committee on the Use of Animals of the institution (23091.011744/2017‐59).

## Owner informed consent

A representative of Apodi‐RN‐Brasil Animal Protection Association (APA) consented to the use of the animals in the research.

## Supporting information

Table S1

## Data Availability

The data that support the findings of this study are openly available in figshare at http:// doi.org /10.6084/ m9.figshare.8429111.
