# Supplementary material for: Post‐operative pain behaviour associated with surgical castration in donkeys (Equus asinus)
Source: Equine Vet J. 2020 Jul 8;53(2):261–6. doi: 10.1111/evj.13306 (PMC7891375; doi:10.1111/evj.13306)
Supplement: Supplementary file 1 — Table S1 [file EVJ-53-261-s001.pdf]

Equine Veterinary Journal BEVA

TABLE S1: Behaviours observed in the videos during the pre-operative period of four donkeys submitted to surgical castration (pilot study) and their description.

**Behaviours**	**Description of behaviours**
Braying	The animal emits a braying sound
Defecation	The animal defecates
Ear movement	The animal moves the ears forward and/or backward, and/or laterally
Eating †	The animal is feeding
Expose penis	The animal exposes its penis without urinating
Extends pelvic limbs	The animal extends both pelvic limbs and remains like this for a few seconds
Flank watching or staring	The animal turns its head to its abdomen
Flexes thoracic limbs	The animal flexes one of its thoracic limbs
Head shaking	The animal shakes its head to both sides
Head turning	The animal turns its head slowly to the sides
Interacts with animal in another stall	The animal places its head over the stall wall and tries to contact another animal
Investigates the stall door	The animal touches its nostrils on the door of the stall
Kicking	The animal makes a kicking movement without being threatened
Lifts pelvic limb	The animal lifts one of the pelvic limbs
Looks for food	The animal lowers its head and looks for food
Movement around the stall†	The animal walks freely in the stall
Pawing at the ground	The animal flexes one of its thoracic limbs and drags the hull on the floor
Position in the stall	Standing near the door, in the front, in the middle, or at the back of the stall
Scratch torso with head	The animal bends and scratches any part of its chest or abdomen with its head
Scratches head on feed trough	The animal rubs its head on the food trough
Scratches head on the wall	The animal rubs its head on the wall of the stall
Scratches head with pelvic limb	The animal scratches its head with one of its pelvic limbs
Scratches testicular area	The animal lowers its head and directs it to the testicular area
Scratches thoracic limb with head	The animal lowers its head and scratches one of its thoracic limbs with its head
Tail swishing	The animal shakes its tail
Urination	The animal urinates
Water drinking	The animal drinks water
Yawn/Flehmen response	The animal opens its mouth making the gesture of yawning or the Flehmen reflex
